# Effectiveness of the Ophthalmological Training Component of the STOMP (Simulation Training for Operational Medical Providers) Curriculum

**DOI:** 10.7759/cureus.13269

**Published:** 2021-02-10

**Authors:** Michael T Spooner, Donald Delorey, Kyle E Miller, Joy A Greer

**Affiliations:** 1 Cardiology, Naval Medical Center Portsmouth, Portsmouth, USA; 2 Psychology, Naval Medical Center Portsmouth, Portsmouth, USA; 3 Ophthalmology, Naval Medical Center Portsmouth, Portsmouth, USA; 4 Obstetrics and Gynecology, Naval Medical Center Portsmouth, Portsmouth, USA

**Keywords:** simulation, ophthalmological procedures

## Abstract

Introduction: Simulation has been used in medicine to train clinicians to manage a variety of clinical scenarios. A key adaptation of the use of simulation in military healthcare occurred in 2015 with the development of the STOMP (Simulation Training for Operational Medical Providers) curriculum, a specific curriculum designed for the intern (PGY-1) trained physicians being sent into the military to practice primary care. Despite showing the curriculum’s influence on self-perceived comfort scores, no study has determined whether simulation is an effective means of improving general medical officer (GMO) physicians’ skills compared to other traditional styles of education. Specifically, this study sought to determine whether simulation-based education (SBE) of ophthalmologic skills improves GMO physicians’ clinical performance, as compared to traditional didactic-based instruction.

Methods: The study, conducted at Naval Medical Center Portsmouth, included GMO physicians who were enrolled in the 2019 STOMP class. Following a brief overview of the study, GMO physicians who elected to participate in the study were randomized to either SBE or lecture-based training for three commonly used ophthalmological procedures: slit lamp exam, tonometry, and corneal foreign body removal. After completing the simulation and lecture-based education training sessions, participants' procedural performance was evaluated utilizing a locally developed performance checklist, and completion time for each of the three procedures was recorded. Data were analyzed using the t-test and Mann-Whitney test. A significance level of 0.05 was considered to be statistically significant.

Results: Of the 50 consented participants, 46 completed the study. The mean overall completion scores for the performance checklists were significantly higher for the SBE group (n=26) compared to the lecture group (n=20) [80% (95% CI 78-82%) vs 41% (95% CI 35-47%), respectively]. Time to completion of the individual tasks was also significantly shorter for the SBE group compared to the lecture group (with mean differences ranging from 27 to 126 seconds, all p<.05).

Conclusions: Simulation-based training appeared to be more effective at teaching three ophthalmological procedures (slit lamp exam, tonometry, and corneal foreign body removal) to GMO physicians compared to didactic-based instruction alone.

## Introduction

Simulation-based education (SBE) is becoming increasingly accepted as a method to complement more traditional clinical training methods in healthcare education [1]. Simulation affords the learner the unique opportunity to push boundaries, and experience and appreciate mistakes, all while receiving feedback in a safe, monitored setting [2]. This process helps then to increase learner confidence. SBE is particularly beneficial for exposure to low-frequency medical scenarios [3]. Although SBE seems logical and has shown great benefit in other fields such as aviation, research demonstrating whether an SBE curriculum is superior to a traditional teaching methods in teaching ophthalmology skills to primary care physicians has not been investigated.

In 2015, a medical simulation physician-based curriculum titled STOMP (Simulation Training for Operational Medical Providers) was developed at Naval Medical Center Portsmouth (NMCP), Virginia, to ensure that general medical officer (GMO) physicians deploying to an austere environment were proficient in the performance of their low-frequency primary care skills. The curriculum was particularly timely, as more than 50% of Navy interns (graduating PGY-1 physicians) provide primary care to operational military forces as GMO physicians [4].

Previous research indicated that the STOMP training was well received by the GMO physician target audience and significantly improved learner’s self-reported confidence levels [5]. However, no research has explored the efficacy of the simulation training of the STOMP curriculum, itself. Based upon greatest change in confidence prior to and after the administration of the curriculum, two of the eight specialty areas of the STOMP Curriculum were identified as possible targets to assess training of clinical skills: Ophthalmology and Orthopedics. The other specialty areas of the STOMP curriculum include: Dermatology, Emergency Medicine, Otolaryngology, General Surgery, Gynecology, and Podiatry. Initially both subjects were targeted for study, but due to difficulty in study execution, only ophthalmology was subsequently pursued for study. A prospective, randomized study was then pursued to determine whether SBE is superior to traditional didactic lecture training. The authors hypothesized that the participants who receive SBE will statistically outperform those completing didactic training as judged by the accurate and efficient (shorter) completion of clinical checklists in the three skill areas associated with the ophthalmological training module.

## Materials and methods

Study design

This study employed a randomized design involving future GMO physicians who participated in the June 2019 STOMP training at NMCP. All physicians were active-duty Navy physicians who had just completed the first post-graduate year (PGY-1) of Accreditation Council for Graduate Medical Education (ACGME)-approved residency training in pediatrics, general surgery, emergency medicine, internal medicine, psychiatry, or a transitional internship. The study was approved by the Naval Medical Center Portsmouth Institutional Review Board and participants were provided informed consent and the ability to opt out of the study. Non-participants still received all of the simulation training associated with the STOMP curriculum, but did not complete the assessment portion.

After obtaining informed consent and prior to the administration of the STOMP training curriculum, participants were randomized to either the SBE or lecture group. The simulation group learned to assess and manage three ophthalmology skills (slit lamp exam, tonometry, and corneal foreign body removal) using instructor proctored simulation, while the lecture group learned to assess and manage the same three ophthalmology skills utilizing a 60-minute lecture format. 

All sessions were proctored by two board-certified staff ophthalmologists who had broad experience in the skills. To ensure quality of the teaching sessions and reliability of the assessment tools, both ophthalmologists were present during the instruction and evaluation. Setup of the ophthalmologic simulation station is described in Appendix A. 

Immediately upon completion of the session, one of the NMCP staff ophthalmologists assessed the learner’s performance real-time during a summative simulation exercise, as measured by a performance checklist that evaluated the participant’s ability to assess and manage an ophthalmological scenario that addressed the three specific skills. No additional study time between instruction and testing was allowed for either the simulation or the lecture group. This study utilized three learning outcomes for participants: 1) mean overall performance checklists scores, 2) performance checklist questions, and 3) simulation scenario completion time, utilizing a stopwatch during testing.

**Figure 1:**
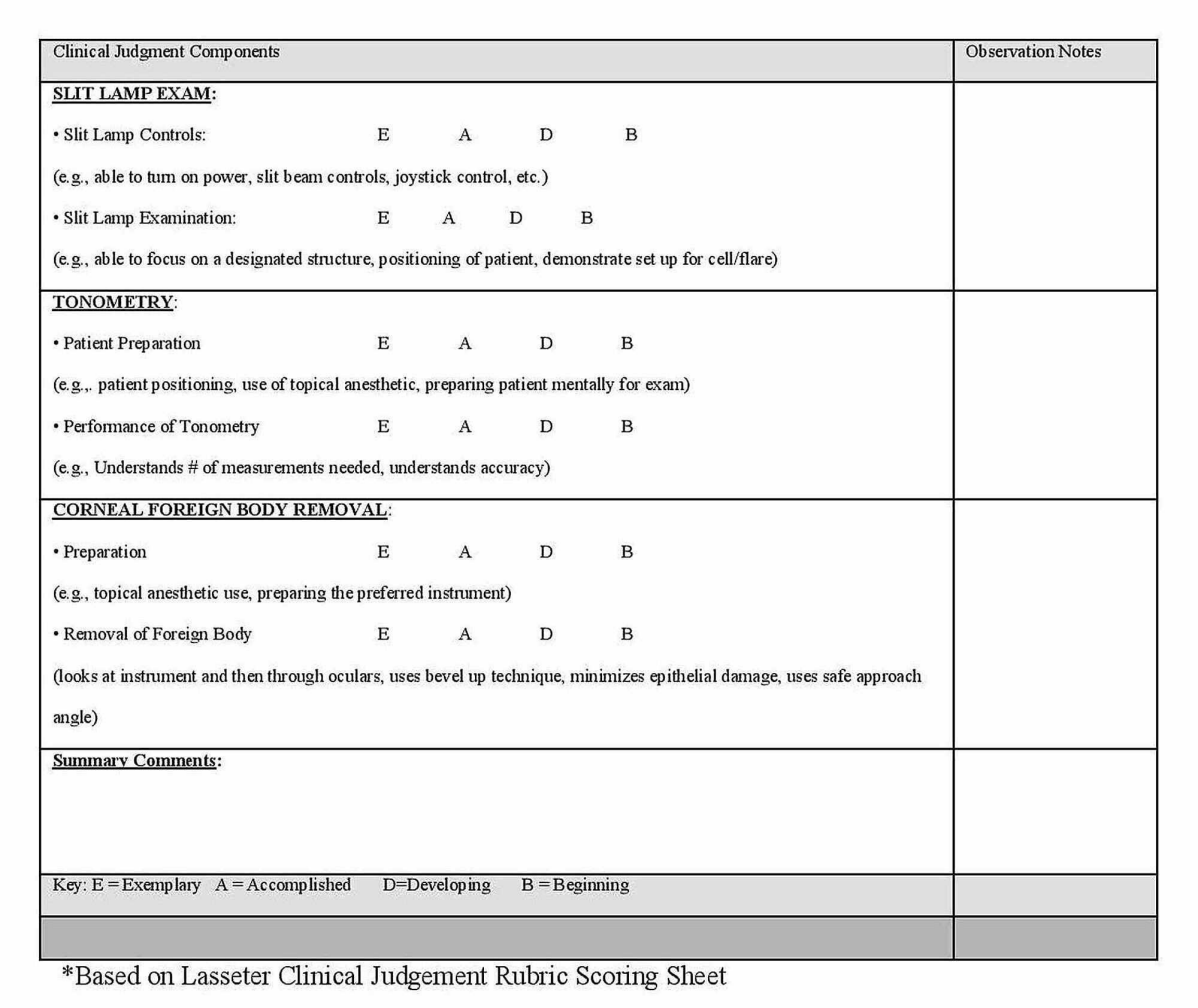
Sample STOMP Ophthalmology Clinical Scoring Sheet

The ophthalmology simulation case scenario and associated performance checklist were developed by the study’s authors in consultation with staff ophthalmologists. The checklist (Figure 1) was based on the Lasseter Clinical Judgment scale, while the lecture material for the training sessions was provided by the individual ophthalmology proctors. All materials and assessment methods were reviewed and approved by the principal investigator prior to the start of the study.

Participants

PGY-1 trained active-duty Navy physicians who had been selected for assignments as GMOs were enrolled in the June 2019 STOMP training program. On the first day of the curriculum in June 2019, participants were recruited for the study and then randomized into two groups.

Statistical analysis 

Ophthalmological performance checklists scores were converted to a percentage of possible points (100 total points) for an overall score. Means and 95% confidence intervals (CIs) were calculated for each overall score. Scores were then compared between simulation and lecture groups and assessed for normalcy of distribution. As the results were non-parametric, a signed-rank test was employed to determine the statistical difference between groups. Completion times for the ophthalmology simulation sessions were analyzed using a t-test for two independent means. A p-value of <.05 was considered significant.

## Results

Of the 51 eligible GMO physicians, 50 STOMP participants consented to participate in the study. All participants selected for the intervention group completed the study. Four individuals in the lecture training withdrew from the study before completing the summative evaluation, leaving 20 participants in the control group for analysis. Demographically, study participants in the two groups were all new PGY-1 graduates from a variety of internships: emergency medicine, internal medicine, general surgery, pediatrics, psychiatry, obstetrics/gynecology, and transitional. Table 1 presents the distribution of specialties amongst the control and intervention groups. 

**Table 1:** Medical Specialties Distribution between the Control and Intervention Groups OB/GYN: Obstetrics and Gynaecology

	Emergency Medicine	Internal Medicine	General Surgery	Pediatrics	Psychiatry	OB/GYN	Transitional
CONTROL (N=20)	1 (5%)	5 (25%)	2 (10%)	1 (5%)	1 (5%)	0%	10 (50%)
INTERVENTION (N=26)	3 (12%)	6 (23%)	4 (15%)	3 (12%)	0%	1 (4%)	9 (35%)

For our primary outcome, the mean overall completion scores for the performance checklists were 80% (95% CI 78-82%) for the SBE group and 41% (95% CI 35-47%) for the lecture group. Simulation training resulted in a 39% absolute increase in scores (95% CI 32-46%).

For our secondary outcome, individual questions on the two performance checklists were also analyzed for significance via the Mann Whitney test. Results indicated that simulation-trained participants scored higher on all three skill components of the ophthalmology assessment compared to the lecture group (p < .05). Table 2 presents the performance checklists findings.

**Table 2:** Ophthalmology Performance Checklist Results SIM: Simulation, LEC: Lecture

OPHTHALMOLOGY - ANALYSIS	AVG SIM SCORE (n=26)	AVG LEC SCORE (n=20)	p
Slit Lamp Exam - clinical judgment component 1			
Question 1 - Controls Manipulation	3.23	1.25	< .00001
Question 2 - Examination	3.19	1.35	< .00001
Tonometry - clinical judgment component 2			
Question 1 - Preparation	3.19	2.6	0.00219
Question 2 - Performance	3.23	2	< .00001
Corneal Foreign Body Removal - clinical judgment component 3			
Question 1 - Preparation	3.35	1.53	< .00001
Question 2 - Removal	3.31	1.05	< .00001
	scale 1-4	scale 1-4	

Participants in the simulation-based training sessions required significantly less time to assess and manage all of the checklist tasks than the lecture-based training participants. Table 3 presents the procedural completion times results.

**Table 3:** Ophthalmology Procedural Completion Times SIM: Simulation, LEC: Lecture

	Completion Time (sec)					
OPTHALMOLOGY AREAS	SIM (n=26)		LEC (n=20)			
	M	SD	M	SD	t-value	p
1. Slit Lamp Exam - clinical judgment component 1	149	46	275	72	-6.77386	< .00001
2. Tonometry - clinical judgment component 2	64	21	174	112	-4.75512	0.000011
3. Corneal Foreign Body Removal - clinical judgment component 3	75	25	102	29	-3.28653	0.000999

## Discussion

Based on the results of this study, SBE appears to be superior to didactic based instruction for teaching future GMO physicians how to assess and manage an ophthalmological scenario associated with the STOMP curriculum both in checklist completion rates and time to complete each skill station. As we hypothesized, all of the physicians who received the simulation-based training performed better on the performance checklists than the lecture-based group, with an overall absolute score improvement of 39%. Our results are consistent with a similar study from 2011 that demonstrated an overall score improvement for simulation-based training of 22% for fourth-year medical students [6]. Additionally, completion times for the three ophthalmological areas support the overall score improvement noted above, indicating that the simulation group not only performed the tasks more accurately but also more efficiently. These findings highlight the importance of using an objective measure (performance checklist) to assess the effectiveness of simulation-based training, as opposed to relying on a subjective measure of effectiveness alone.

This is the first study associated with the STOMP curriculum to show objective measurable performance of participants who receive hands-on simulation training. The finding of this study highlights the benefits of simulation training over standard didactic training for developing basic ophthalmic skills prior to GMO assignment. Our results support the more widespread use of SBE in ophthalmology, a specialty that has less commonly employed simulation training technology. Possible explanations include the misperception that ophthalmological topics do not lend themselves as well to a simulation environment as other medical specialties, or a reduced number of simulation educators in the field of ophthalmology. Demonstrating the effectiveness of ophthalmological SBE is a necessary step to move the field forward [7].

Strengths

Strengths of this study include its randomized nature, with an adequate sample size of 46 participants (post hoc power analysis indicated 89% power), and the use of a novel performance checklist to objectively assess each participant’s knowledge and performance. Another strength of the study was its ability to assess outcomes that are “higher” on Miller’s Pyramid, a popular model for assessing clinical competence [8]. The lowest level of the pyramid is knowledge (“knows”), followed by competence (“knows how”), performance (“shows how”), and action (“does”). The ophthalmological simulation training targeted both the “knows how” layer and the “shows how” layer. This study also addressed the concern of additional study time by ensuring control subjects and intervention subjects had the same amount of study preparation time. The authors believe that this policy more accurately reflects the intervention on participant’s performance regardless of the modality - simulation or lecture.

Limitations

Our study had several limitations. As the study was not longitudinal in nature, the benefit of this type of intervention over time could not be determined. Instructors were not blinded to the training modality they were using to teach participants and were the same ophthalmologists used to test the subjects, given the lack of other available ophthalmologists. Also, we did not attempt to determine whether simulation performance translates into improved performance in real clinical settings. While the current standard for preparing General Medical Officers is to not utilize simulation training, it is possible given the psychomotor nature of scenario completion tasks, that the simulation group’s improved performance was influenced by their ability to practice and rehearse these skills before testing. Finally, as the assessment occurred immediately after the teaching sessions, we are not able to determine which teaching method leads to better long-term skills retention.

## Conclusions

In conclusion, the results of this study supports other research which indicates that simulation-based training is superior to didactic lecture in teaching a skills- and knowledge-based clinical competency. We specifically show the value and importance of simulation-based training for PGY-1 trained physicians in the assessment and management of three ophthalmological clinical tasks prior to their assignment as GMOs.

## Appendices

SLIT LAMP EXAM:

1. The learner can utilize slit lamp controls. 

A. The learner can turn on power.  The learner understands how to change oculars for examiner interpupillary distance.                                              

B. The leaner can control and change slit beam.  Learner understands how to measure using a slit lamp beam, how to utilize an oblique slit lamp beam, and how to reduce slit beam glare.

C. The learner knows how to use joystick controls.  The learner understands how to adjust magnification of the slit-lamp.

D. The learner knows and can describe basic eye anatomy

2. The learner can perform slit Lamp Examination

A. The learner is able to focus on a designated structure

B. The leaner can properly position the patient                                                                                                                                                           

C. The learner can demonstrate set up for cell/flare

TONOMETRY: 1. The leaner can correctly prepare the patient for TonoPen Tonometry. 

A. The learner properly positions the patient

B. The learner uses topical anesthetic properly

C. The learner prepares the patient for the examination

D. The learner understands which patient not to perform test on - i.e. Open-globe injury

E. The learner understands how to calibrate the instrument

F. The learner understands the use of disposable covers to prevent contamination

2. The learner can correctly perform TonoPen Tonometry

A. The learner understands the number of measurements used

B. The learner can interpret the accuracy of the test

C. The learner understands the normal range of intraocular pressure

CORNEAL FOREIGN BODY REMOVAL:

1.  The learner can properly prepare the patient for foreign body removal.

A. The learner applies appropriate topical anesthetic

 B. The leaner prepares the preferred instrument for removal (CTA, etc).

2.  The learner can correctly remove a Foreign Body

A. The learner looks at instrument through oculars

B. The learner uses bevel up technique and uses safe approach angle

C. The leaner minimizes epithelial damage
